# Letter from the Editor in Chief

**DOI:** 10.19102/icrm.2022.130907

**Published:** 2022-09-15

**Authors:** Moussa Mansour


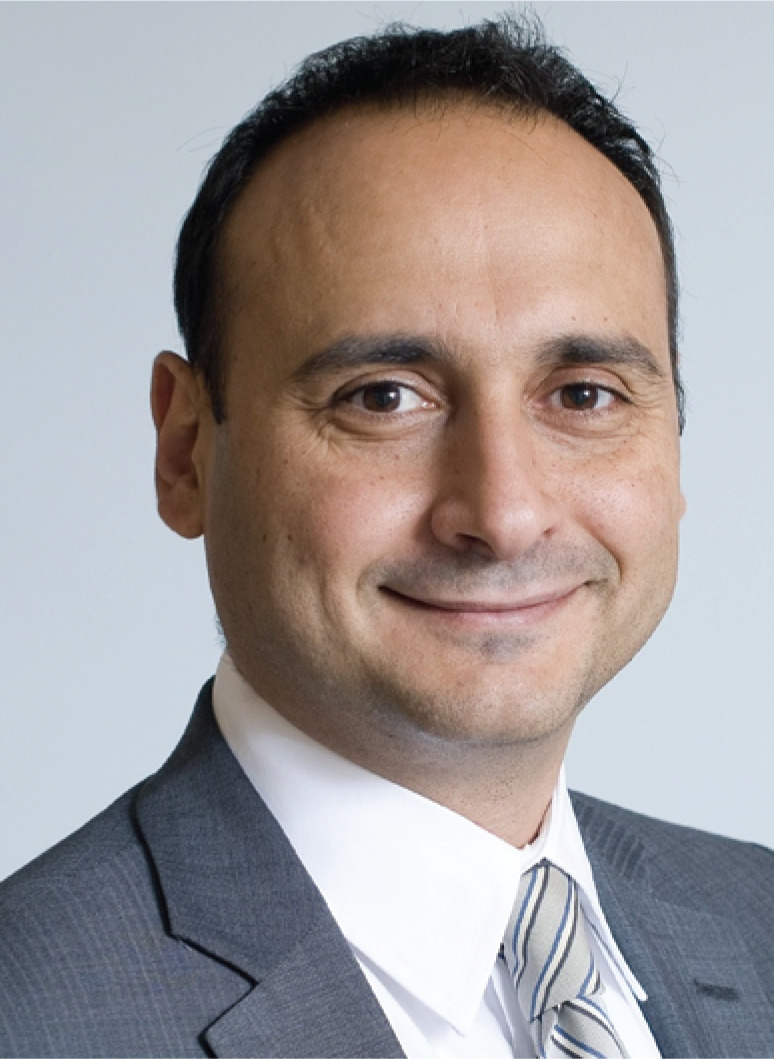


Dear readers,

The annual scientific meeting of the European Society of Cardiology (ESC) was held recently in Barcelona. Many studies in the field of cardiac electrophysiology were discussed, including the Catheter Ablation for Persistent Atrial Fibrillation (AF): A Multicenter Randomized Trial of Pulmonary Vein Isolation (PVI) Versus PVI with Posterior Left Atrial Wall Isolation (PWI) (CAPLA) study, which was presented by Dr. Peter Kistler. In the study, 338 patients with persistent AF were randomized to either PVI alone or in combination with PWI. The study endpoint was freedom from AF for >30 s, which was reached in 52.4% of cases in the PVI-alone group compared to 53.9% of cases in the PVI+PWI group, with a hazard ratio of 1.01 (95% confidence interval, 0.74–1.38, *P* = .96). The authors concluded therefore that PWI does not improve clinical outcomes in patients with persistent AF.

This study is intriguing because its results contradict a large number of prior data demonstrating the incremental value of ablation of the left atrial posterior wall in patients with AF. The posterior left atrial wall has the same embryological origin as the pulmonary veins^1^; as such, one would expect both that it is likely to trigger AF the same way the pulmonary veins do and that including it in the PVI lesion set would improve the outcome of ablation. This rationale has been demonstrated in numerous randomized and non-randomized clinical studies pioneered by Dr. Andrea Natale >20 years ago and was recently summarized in a meta-analysis published in 2020.^2^ These earlier studies were conducted using both radiofrequency energy and cryothermy. Moreover, a recent ablation study using pulsed-field ablation to treat the posterior wall resulted in excellent success rates in patients with persistent AF.^3^ The findings in all of these studies are consistent.

The findings of the CAPLA study have not been published yet and were only presented at ESC 2022 in abstract form. As a result, it may be too early to fully understand the study’s results. There are, however, a few points that might explain its findings. First is the technique of isolation of the posterior wall. One would imagine that isolation of the posterior wall and pulmonary veins in a box lesion set might not be as effective as ablating it and pacing to prove the rigor of isolation. The technique of isolation may also explain the success rate in the CAPLA study, which was remarkably lower than rates in other ablation studies that enrolled similar populations, such as the Safety and Effectiveness of the THERMOCOOL SMARTTOUCH^®^ SF Catheter Evaluated for Treating Symptomatic Persistent AF (PRECEPT) study,^4^ where the success rate was significantly higher and defined using a very stringent monitoring regimen. Another factor to consider is the number of enrolled patients. The calculated study population in CAPLA was small, likely secondary to the hypothesis of the authors, who assumed that ablation of the posterior wall would result in an additional 15% benefit, which is significantly higher than what is presumed by most studies investigating the role of ablation of the posterior left atrial wall. A smaller assumed difference would have required a larger study and could have resulted in a different outcome. The number of patients enrolled in CAPLA was also smaller than that in studies comparing PVI alone to PVI plus adjunctive ablations. Both the Efficacy of Delayed Enhancement MRI-guided Ablation vs. Conventional Catheter Ablation of AF (DECAAF II) study^5^ that compared PVI alone to PVI plus ablation of fibrotic areas and the LAA Ligation Adjunctive to PVI for Persistent or Longstanding Persistent AF (aMAZE) trial^6^ that compared PVI alone to PVI plus ligation of the left atrial appendage enrolled close to 1,000 patients each.

In summary, the rationale based on the embryologic origin of the posterior wall of the left atrium being an extension of the pulmonary veins and the large body of evidence derived from many ablation studies support the benefit of ablating the posterior wall in addition to performing PVI in patients with AF. Moreover, with the imminent introduction of pulsed-field ablation, I believe that the ablation of the posterior wall will become easier and safer to perform and that PVI and PWI together will likely become a cornerstone of AF ablation in all patients.

I hope that you enjoy reading this issue of *The Journal of Innovations in Cardiac Rhythm Management*.


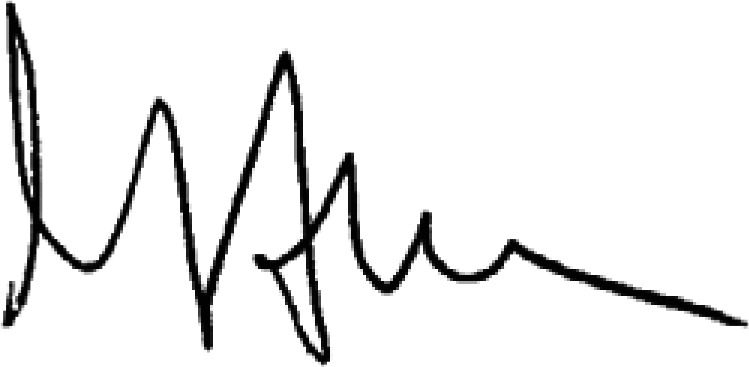


Sincerely,

Moussa Mansour, md, fhrs, facc

Editor in Chief

*The Journal of Innovations in Cardiac Rhythm Management*

MMansour@InnovationsInCRM.com

Director, Atrial Fibrillation Program

Jeremy Ruskin and Dan Starks Endowed Chair in Cardiology

Massachusetts General Hospital

Boston, MA 02114
